# A rare cause of specific cough in a child: the importance of following-up children with chronic cough

**DOI:** 10.1186/1745-9974-1-8

**Published:** 2005-09-21

**Authors:** Richard Lloyd Barr, David John McCrystal, Christopher Francis Perry, Anne B Chang

**Affiliations:** 1 Senior Resident, Royal Children's Hospital, Herston Rd, Brisbane, Qld 4029, Australia; 2 ENT Registrar, Royal Children's Hospital, Herston Rd, Brisbane, Qld 4029, Australia; 3 Consultant in ENT Surgery, Royal Children's Hospital, Brisbane; Herston Rd, Brisbane, Qld 4029, Australia; 4 Consultant Respiratory Physician, Dept of Respiratory Medicine, Royal Children's Hospital, Brisbane; Herston Rd, Brisbane, Qld 4029, Australia; and A/Professor of Paediatrics, University of Queensland, Herston Rd, Brisbane, Australia

## Abstract

For many years, the term 'specific cough' has been used as a clinical cough descriptor in children to signify the likelihood of an underlying disease causing the cough. In this case study, we describe a child with specific cough caused by a rare carcinoma, a mucoepidermoid carcinoma of the bronchus. The cough only totally resolved after the primary cause was successfully treated. This report highlights the importance of following up children with cough, especially those with specific cough.

## Clinical Record

An 8-year-old girl from a remote Aboriginal community approximately 2500 km from Brisbane was transferred to our hospital for management of a bronchial lesion. She had received 7-days of intravenous amoxicillin prior to transfer. She had a 4-year history of daily wet and sometimes productive cough, which was worse on exertion. There was no history of exertional dyspnoea, haemoptysis or weight loss. She also had a history of recurrent admissions for pneumonia at the local hospital (3 in the past 6 months). In the child's community, two adults were recently diagnosed with active pulmonary tuberculosis.

**Figure 1:**
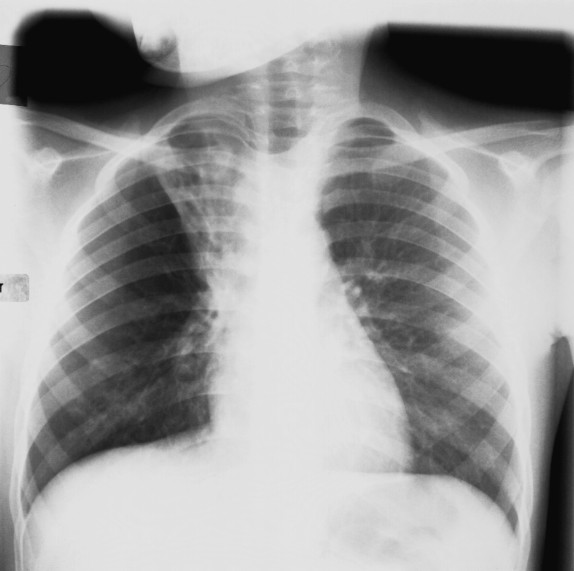
Chest x-ray of the child 4 months before referral. The CXR shows collapse and tram tracks of the right upper lobe and increased peribronchial and interstitial markings of the right lower lobe.

**Figure 2:**
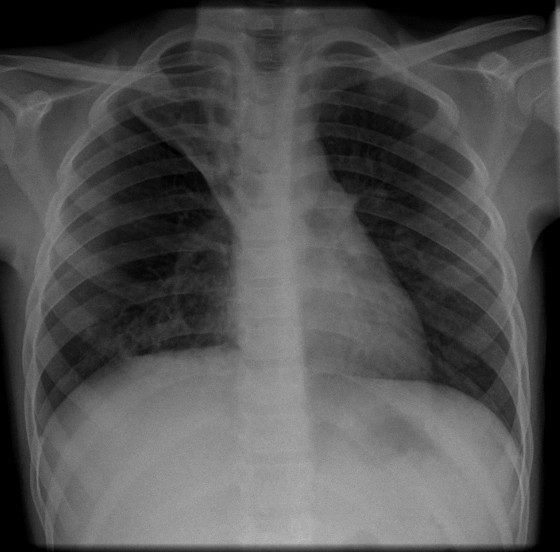
CXR of child from referral hospital showing minimal increased changes from CXR taken 4 months ago.

**Figure 3:**
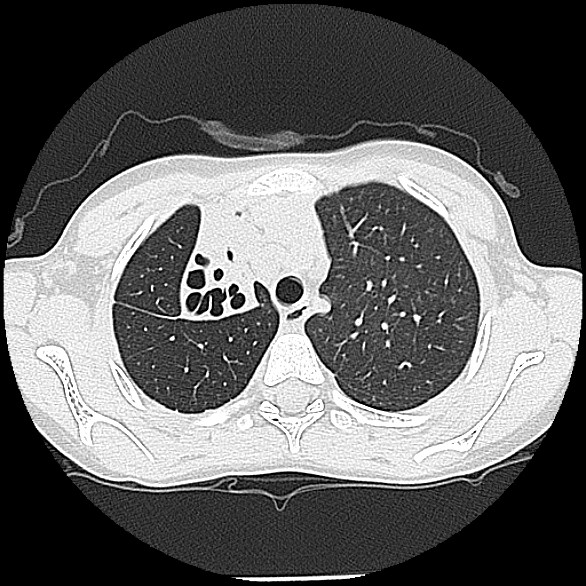
Representative high resolution CT chest slices demonstrating collapse and severe bronchiectasis of the right upper lobe.

**Figure 4:**
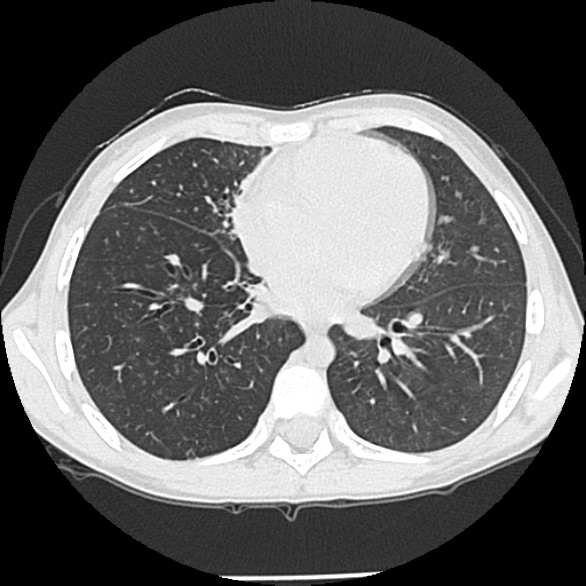
Representative high resolution CT chest slices demonstrating 'mild' bronchiectasis of the right lower lobe with partial collapse of right middle lobe. Bronchiectasis also present in the right middle lobe is not clearly demonstrated here.

**Figure 5:**
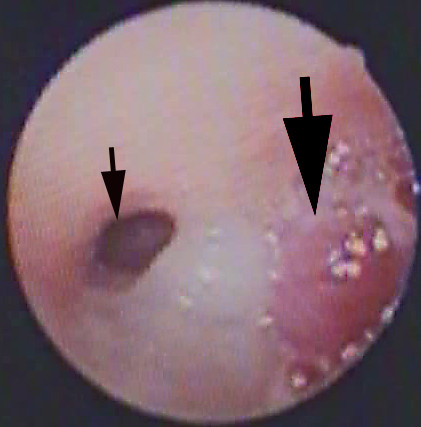
Bronchoscopic picture of the carina prior to bronchoscopic partial removal of the tumour. The mucoepidermoid carcinoma that arose from the right upper lobe bronchus was so large it protruded into and obstructed the entire right main stem and is clearly visible at the carina (large arrow). The left main bronchus (small thick arrow) is partially occluded by secretions.

On arrival, the child was thin (weight 5^th ^percentile, height 25^th^), appeared well and had a wet cough, reduced air entry over the right side and inspiratory crepitations. Spirometry values were invalid as she could not adequately perform maximum expiratory manoeuvres. Chest x-ray (CXR) showed right upper lobe (RUL) collapse, tram-tracks signs and increased peribronchial and interstitial markings of the right lower lobe. These CXR changes were documented at least 4-months ago (figures 1 and 2). Chest high resolution computerised tomography (CT) scan revealed RUL collapse and severe cystic bronchiectasis and cylindrical bronchiectasis of the right middle and lower lobes (figures 3 and 4). Sputum cultures grew *Moraxella catarrhalis*, and the microscopy was negative for acid-fast bacilli. Mantoux tests (*M. tuberculum, M. Avium*) were negative, sweat test and immunological workup were normal. Flexible bronchoscopy revealed a large lesion at the carina (Figure 5). Rigid bronchoscopy was then immediately performed during which the lesion was only partially removed piecemeal because of the presumed diagnosis of tuberculosis and length of time required to remove the bulk of the lesion (2-hours). Given the significant tuberculosis contact, anti-tuberculous medications were commenced and later ceased when cultures and Quantiferon test were negative. Histology showed a subepithelial neoplasm comprising glandular and solid areas with no evidence of significant mitotic activity or atypia, consistent with a low-grade muco-epidermoid carcinoma (MEC). Cytogenetic investigation on the tumour was not performed. Chest and abdomen CT scans revealed no metastases. Bronchoscopy was repeated and the remaining small lesions were biopsied. Right upper lobectomy and lymph node sampling was then performed and histological examination of the operative specimen demonstrated a small amount of residual tumour (with clear resection margins) and bronchiectasis. No metastases were found in the sampled lymph nodes. Postoperative progress was uneventful and the child was discharged 9-days later and was cough free. When reviewed 4 months post-discharge, she remained cough free and a repeat flexible bronchoscopy then confirmed the absence of any bronchial lesion or secretions.

## Discussion

We have described a child with several features of chronic specific cough caused by suppurative lung disease secondary to a rare life threatening lesion, a mucoepidermoid carcinoma obstructing a major bronchus. The child's cough only totally resolved upon removal of the tumour; i.e. after the primary cause was successfully treated. This report illustrates the importance of following-up children with chronic cough. Cough was this child's only symptom that was consistently present between the child's recurrent hospitalisations.

Paediatric cough, unlike cough in adults, is generally classified for practical purposes into cough descriptors of 'non-specific' and 'specific' cough [1,2]. In children with wet cough, airway secretions are always present [3]. Wet cough is a feature of specific cough as children (especially young children), unlike adults, do not often expectorate sputum. Several features of specific cough were present in this child; specifically, daily moist or productive cough, recurrent pneumonia and abnormal auscultatory findings [1] were present. Thus she had specific cough pointers and, in ideal circumstances, clinicians would be cognisant that the cough is likely associated with an underlying respiratory problem and hence requires further workup and follow-up to define the aetiology. Also, in children, the recommended minimum investigations for any child with a chronic cough are a CXR and spirometry [4]. In this child, the CXR was clearly abnormal – another indicator that further follow-up and investigations are usually required. This child had clinical features of bronchiectasis for at least several months and most likely a few years before eventual diagnosis of the underlying cause of her cough and respiratory illness. Also, radiological evidence of bronchiectasis was present and was secondary to a low-grade MEC that caused obstructive bronchiectasis (hence chronic wet cough from suppurative lung disease) and recurrent pneumonia. Unfortunately, the bronchiectasis was not restricted to the RUL; the delay in diagnosis allowed growth of the tumour that was so large it obstructed the entire right main bronchus and lead to obstructive bronchiectasis of the right lung.

**Figure 6:**
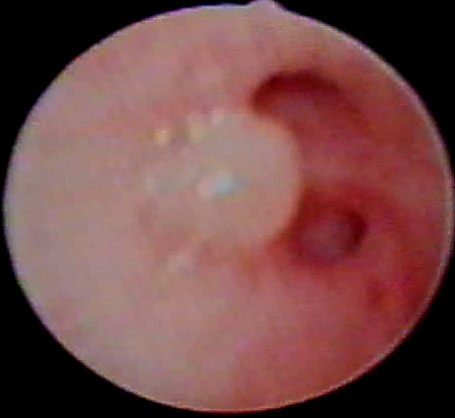
Figure showing bronchial non-tuberculous mycobacterium lesion of right upper lobe subsegment from another child. Macroscopically MEC appear similar to bronchial tuberculosis and can only be confidently differentiated by histopathology. This non-indigenous child presented with a few months history of chronic cough.

Lung carcinoma remains the most common cancer in adults but is very rare in children [5]. Pulmonary MEC are even more rare (only 53 paediatric reports) [6-8] and represent approximately 10% of paediatric pulmonary tumours [7]. Macroscopically, MEC appear as a polypoid mass extending into the lumen [6-9] which may appear similar to bronchial mycobacteria lesions (Figure 6). Definitive diagnosis requires tissue biopsy, usually taken at bronchoscopy [6,7]. Because MEC are covered by normal respiratory epithelium bronchial brushings are usually not diagnostic [7,10]. MEC is thought to arise from mucous glands in the submucosal layer of respiratory walls [8,11] and is phylogenetically similar to salivary gland tumours [10]. Cytogenetic analysis of MEC tumours have described the presence of translocation t(11;19) (q14-21;p12-13) [12]. MEC has an 'iceberg-like' tendency to extend partially into the airway lumen but may extend into surrounding lung parenchyma [7]. Histologically, these tumours consist of a mixture of epidermoid, mucous and intermediate cells and may be classified as low, intermediate or high grade, reflecting differing compositions of cell types, extent of mitosis, anaplasia, and morphological variance ranging from cystic through to solid in nature [7,8,10]. Low grade tumours, more common in children, predominantly consist of mucous cells with occasional intermediate cells, tend to be locally invasive and, are associated with long term survival [9]. Intermediate grade tumours are more solid with predominance of intermediate cells and occasional mucous cells [8]. High grade tumours, more common in adults have a poorer prognosis [6-8,10,11]. with metastatic spread via blood or lymphatics to skin, bone and pericardium [8]. In all but two of the reported paediatric cases including ours, MEC was found to be low grade, and these tumours were successfully resected with no recurrence on follow up [7,8]. Children with high grade tumour succumb early, with one report of a child with a high grade tumour who succumbed eight months after diagnosis [7].

Presentation of patients with MEC is unusual until some obstruction of the involved airway occurs [6-9]. Common presenting symptoms include cough, recurrent pneumonia, haemoptysis, wheeze, dyspnoea, fever, and chest pain [7,8,13]. The rarity of these tumours contributes to delays in diagnosis [7,8]. While a diagnostic delay of up to 20-months has been reported [8], the likely several years interval in this child seemed particularly noteworthy. Deficiencies in health resources available in remote regions are well documented [14]. Indigenous Australians comprise a significant subset of this population and are particularly afflicted by respiratory illness [15,16]. As many of the presenting respiratory symptoms have an infective cause, the diagnostic suspicion of carcinoma in this setting is potentially further reduced. While adverse outcomes may be minimal, delays in diagnosis could lead to increased and prolonged morbidity. This report highlights the need to clinically follow-up all children with chronic cough especially those with chronic specific cough. After successful treatment of the underlying cause, cough almost always resolves in children. In patients with chronic specific cough and/or other respiratory symptoms not responsive to standard medical therapy, further investigations that include radiology and, in selected children, bronchoscopy should be promptly initiated [4].
